# Over-expression of PPAR-γ2 gene enhances the adipogenic differentiation of hemangioma-derived mesenchymal stem cells *in vitro* and *in vivo*

**DOI:** 10.18632/oncotarget.23705

**Published:** 2017-12-26

**Authors:** Si-Ming Yuan, Yao Guo, Qian Wang, Yuan Xu, Min Wang, Hai-Ni Chen, Wei-Min Shen

**Affiliations:** ^1^ Department of Plastic Surgery and Vascular Biology Lab, Jinling Hospital, School of Medicine, Nanjing University, Nanjing, Jiangsu 210002, China; ^2^ Department of Plastic Surgery, Children's Hospital, Nanjing, Jiangsu 210008, China

**Keywords:** infantile hemangioma, involution, mesenchymal stem cell, PPAR-γ, adipogenesis

## Abstract

**Background:**

Most of infantile hemangiomas involute into fibrofatty tissue in childhood, which indicates adipogenesis during this period. Mesenchymal stem cells (MSCs) contribute to the adipogenesis in IH. In this study, we investigated the effects of overexpression of PPAR-γ2 gene on the adipogenic differentiation of Hemangioma-derived MSCs (Hem-MSCs), and discussed the possibility of targeted therapy via PPAR-γ pathway.

**Methods:**

MSCs were isolated from proliferating hemangioma by their selective adhesion to plastic culture dishes. Recombinant lentivirus with PPAR-γ2 gene were prepared, and used to transfect Hem-MSCs. Transfected cells were cultured in adipogenic medium to observe the differentiation *in vitro*. And the cells were mixed with Matrigel, then subcutaneously injected into the back of nude mice to observe the differentiation *in vivo*.

**Results:**

In the *in vitro* tests, Hem-MSCs with overexpression of PPAR-γ2 gene showed enhanced adipogenic differentiation with increased expression of adipogenic-related genes, including PPAR-γ2, ADD1, LPL, and CEBPA genes. In the *in vivo* tests, Hem-MSCs/Matrigel plugs with overexpression of PPAR-γ2 gene also showed accelerated adipogenesis and time-phased changes of above genes.

**Conclusions:**

Overexpression of PPAR-γ2 gene enhances and accelerates the adipogenic differentiation of Hem-MSCs *in vitro* and *in vivo*. The results may provide the preliminary evidences for the targeted therapy of IH via PPAR-γ signal pathway.

## INTRODUCTION

Infantile hemangioma (IH) is a common benign tumor among infants, with higher incidence in females. It may grow quickly in six months to one year after birth, and afterwards regresses slowly into fibrofatty tissue in childhood [1]. Although most of IHs tend to regress spontaneously, rapid growth of proliferating hemangioma may result in hypertrophy, hemorrhage, ulceration and scar [2]. IH in certain sites, such as the eyelid and nostril, may do harm to vision and breath [3]. Therefore, early treatment is necessary to control IH's growth, promote its involution, and avoid the complications.

Although some clues have been found out, the mechanism of spontaneous remains unclear till now [4, 5]. Interestingly, fibrofatty tissue accumulates during involuting phase, which indicates adipogenesis in this process. Studies have reported hemangioma-derived mesenchymal stem cells (Hem-MSCs) had adipogenic potential both *in vitro* and *in vivo*, which suggested that MSCs contributed to the adipogenesis in IH [6–9].

Peroxisome proliferator-activated receptor (PPAR) is a nuclear hormone receptor family of transcription factors, including three subtypes, i.e. PPAR-α, PPAR-β, and PPAR-γ [10]. Among them, PPAR-γ plays important role in adipogenesis and lipid storage [11]. It promotes the expression of genes related to adipogenesis via formation of a heterodimeric DNA-binding complex with the retinoid X receptor-α (RXR-α) [12]. PPAR-γ consists of four iso-forms, i.e. PPAR-γ1, PPAR-γ2, PPAR-γ3, and PPAR-γ4. Yu Y et al and our study [6, 8] showed that PPAR-γ2 gene was expressed in Hem-MSCs, which indicated the role of PPAR-γ2 gene in adipogenic differentiation of MSCs in hemangioma. In this study, we investigated the effects of over-expression of PPAR-γ2 gene on adipogenic differentiation of Hem-MSCs and discussed the possibility of targeted therapy via PPAR-γ signal pathway.

## RESULTS

### Hem-MSCs were separated and cultured

**Figure 1:**
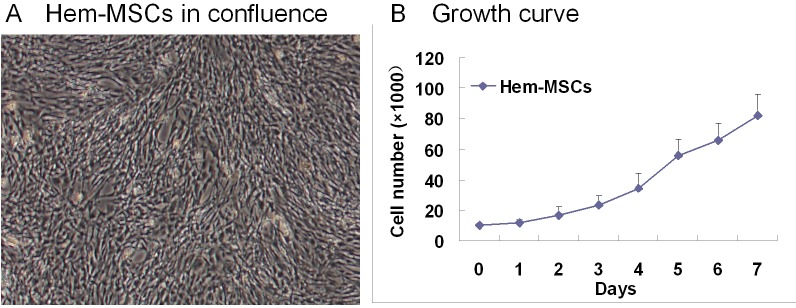
The culture of Hem-MSCs **(A)** the cells in confluence. **(B)** The growth curve.

In primary culture, Hem-MSCs were fibroblast-like and came to confluence in about ten days (Figure 1A). The cells were split at the ratio of 1:3. The growth curve showed that the doubling time of Hem-MSCs was about 2∼3 days (Figure 1B). IHC staining, IF staining, and Flow cytometry showed that the antigen profile and multi-lineage differentiation of Hem-MSCs were consistent with that in our previous report [8].

### Recombinant lentivirus with PPAR-γ2 gene was prepared

**Figure 2:**
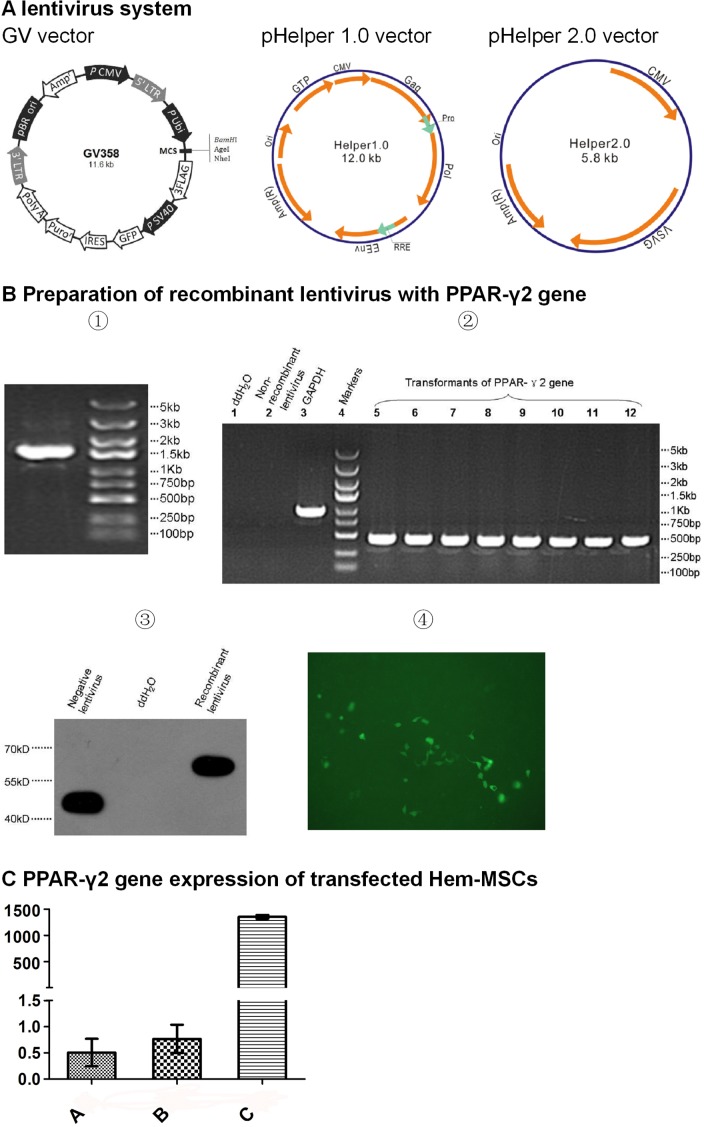
Preparation of recombinant lentivirus with PPAR-γ2 gene **(A)** Lentivirus system. GV358 vector with GFP gene is the core vector to recombine PPAR-γ gene. pHelper 1.0 vector encodes the structural protein. pHelper 2.0 vector encodes envelope protein. **(B)** Preparation of recombinant lentivirus. ①Clones of PPAR-γ2 gene templates. ②Transformants of PPAR-γ2 gene from competent E.coli. ③Expresssion of three flag-PPAR-γ2 fusion protein. ④Measurement of the titer of lentivirus concentration by fluorescence method. This sample pixture was 1E^−1^μL (200x). **(C)** PPAR-γ2 gene expression of Hem-MSCs transfected by recombinant lentivirus. QPCR showed the expression in Group C (recombinational lentivirus+DMEM-LG) was significantly higher than that in Group A (DMEM-LG) and Group B (non-recombinant lentivirus+DMEM-LG) (P<0.01).

After the clone of PPAR-γ2 gene templates, size of the PCR products was examined by running 5μl products on 3% agarose gel. The targeted band appeared between 2kb and 1.5kb, which accorded with the anticipation (1559bp) (Figure 2B①). Positive clone of the competent Escherichia coli transformed by GV core vector with PPAR-γ2 gene was confirmed by PCR (Figure 2B②) and the sequence alignment showed 100% accordance with targeted gene (Supplementary Figure 1). Western blotting showed the expression of three flag-PPAR-γ2 fusion protein in 293T cell transfected by GV core vector with PPAR-γ2 gene, which confirmed the successful recombination of targeted gene (Figure 2B③). After harvesting the lentivirus, fluorescence method was used to measure the titer of the lentivirus concentration (Figure 2B④).

### Transfection of recombinant lentivirus significantly elevated PPAR-γ2 gene expression in Hem-MSCs

Quantitative real time polymerase chain reaction (QPCR) showed the expression of PPAR-γ2 gene in Group C (recombinant lentivirus+DMEM-LG) was significantly higher than that in Group A (DMEM-LG) and Group B (non-recombinant lentivirus+ DMEM-LG) (P<0.01). The gene expression of Group A and Group B is similar (P>0.05). The results indicated that the transfection of the recombinant lentivirus obviously elevated the PPAR-γ2 gene expression in Hem-MSCs (Figure 2C).

### Hem-MSCs with over-expression of PPAR-γ2 gene showed enhanced adipogenic differentiation *in vitro*

**Figure 3:**
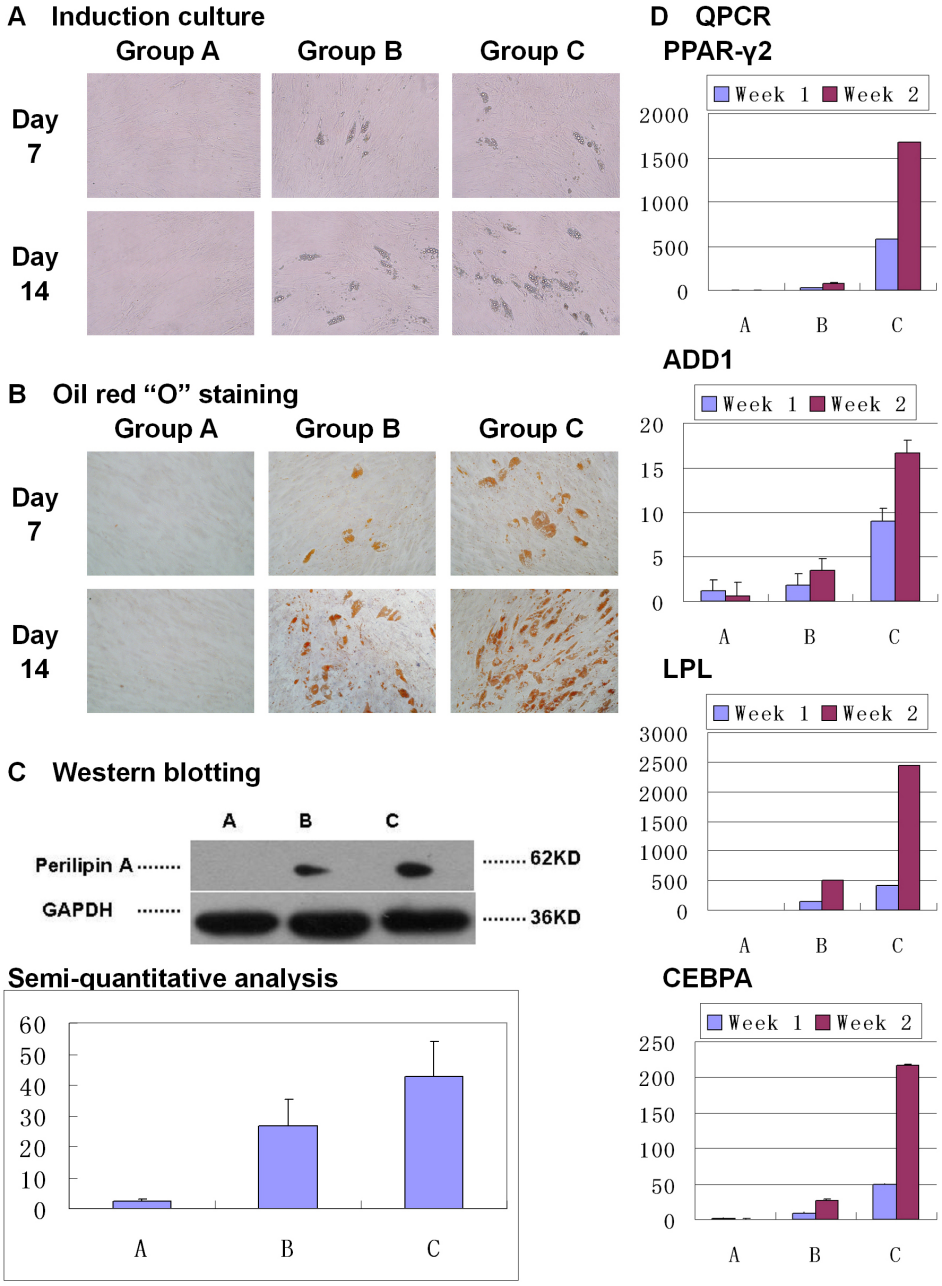
Over-expression of PPAR-γ2 gene enhanced adipogenic differentiation of Hem-MSCs *in vitro* **(A)** Induction culture. In Group A (DMEM-LG), no lipid droplet appeared. In Group B (non-recombinant lentivirus+ DMEM-LG, and then adipogenic medium) and Group C (recombinant lentivirus+ DMEM-LG, and then adipogenic medium), lipid droplets appeared on Day 7 and increased on Day 14, with more in Group C. **(B)** Oil red “O” staining showed the lipid droplets. **(C)** Western blot and semi-quantitative analysis showed obviously higher expresssion of perilipin A in Group C than that in Group B on Day 14 (P<0.05). **(D)** QPCR showed that PPAR-γ2, LPL, ADD1, and CEBPA genes’ expression increased in Group B and C on Day 7 and Day 14 after induction, but not in Group A. And the genes’ expression was obviously higher in Group C than that in Group B on Day 7 and Day 14 (P<0.05).

Seven days after induction, lipid droplets appeared in Group B and Group C, but not in Group A. Fourteen days after induction, lipid droplets in Group B and Group C increased, with obviously more in Group C. No lipid droplet appeared in Group A throughout the culture (Figure 3A). Oil red “O” staining showed the lipid droplets (Figure 3B).

Perilipin A, a marker protein of adipogenic differentiation, was expressed in Group B and Group C 14 days after induction, but did not appear in Group A. Semi-quantitative analysis showed that there was obviously higher expression in Group C than that in Group B (Figure 3C, P<0.05).

PPAR-γ2, LPL, ADD1, and CEBPA genes’ expression was increased in Group B and C on 7 days and 14 days after induction, but not in Group A. And above genes’ expression was obviously higher in Group C than that in Group B (Figure 3D, P<0.01).

### Hem-MSCs with over-expression of PPAR-γ2 gene showed enhanced adipogenic differentiation *in vivo*

**Figure 4:**
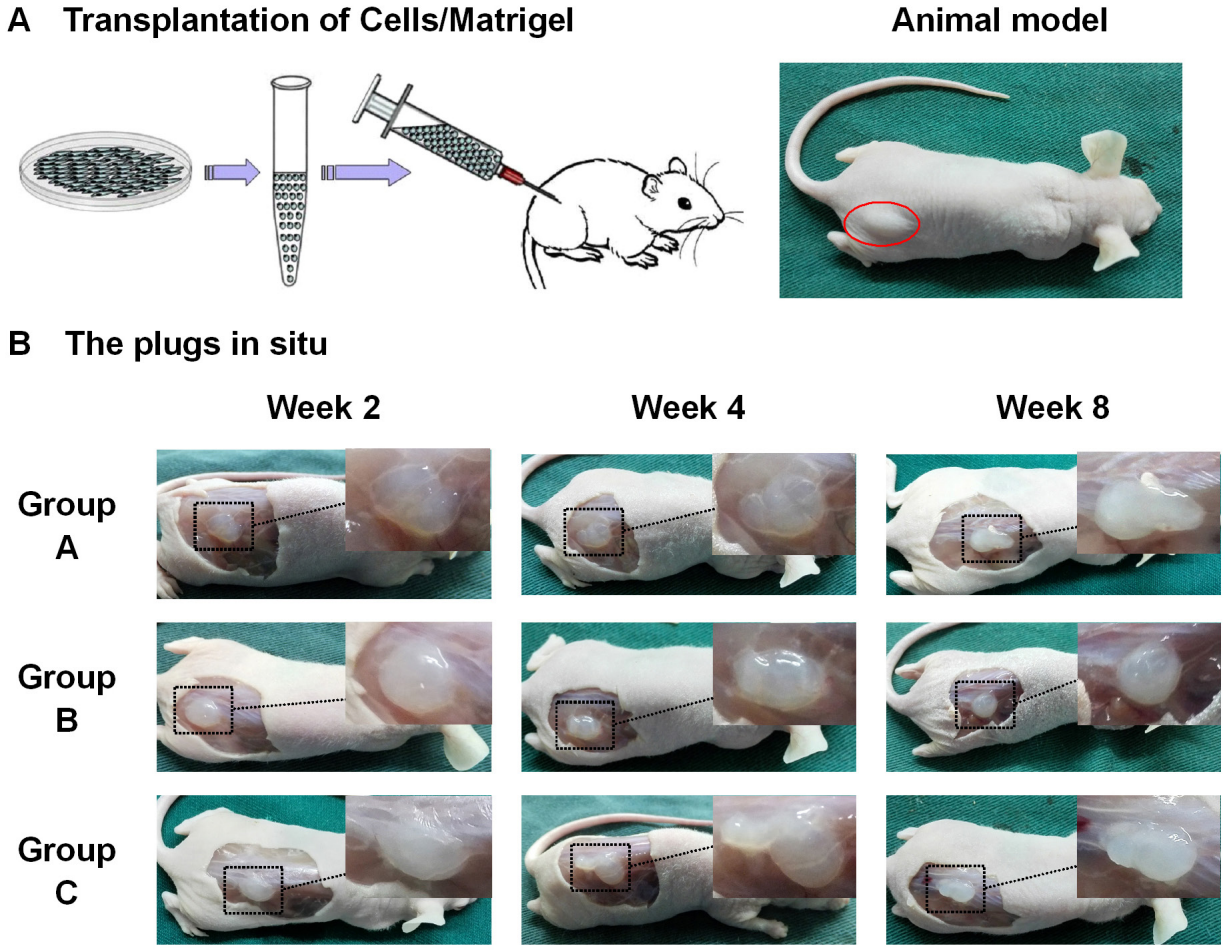
Establishment of *in vivo* model of adipogenic differentiation of Hem-MSCs **(A)** Hem-MSCs were mixed with Matrigel and subcutaneously injected into the back of nude mice. **(B)** The cells/Matrigel plugs were taken out two, four, and eight weeks after injection.

*In vitro* tests confirmed overexpression of PPAR-γ2 gene enhanced adipogenic differentiation of Hem-MSCs. In order to observe the effects *in vivo*, Hem-MSCs tranfected by recombinant lentivirus were mixed with Metrigel and subcutaneously injected into the back of nude mice (Figure 4A). The cells/Matrigel plugs were taken out at two, four and eight weeks after injection (Figure 4B).

**Figure 5:**
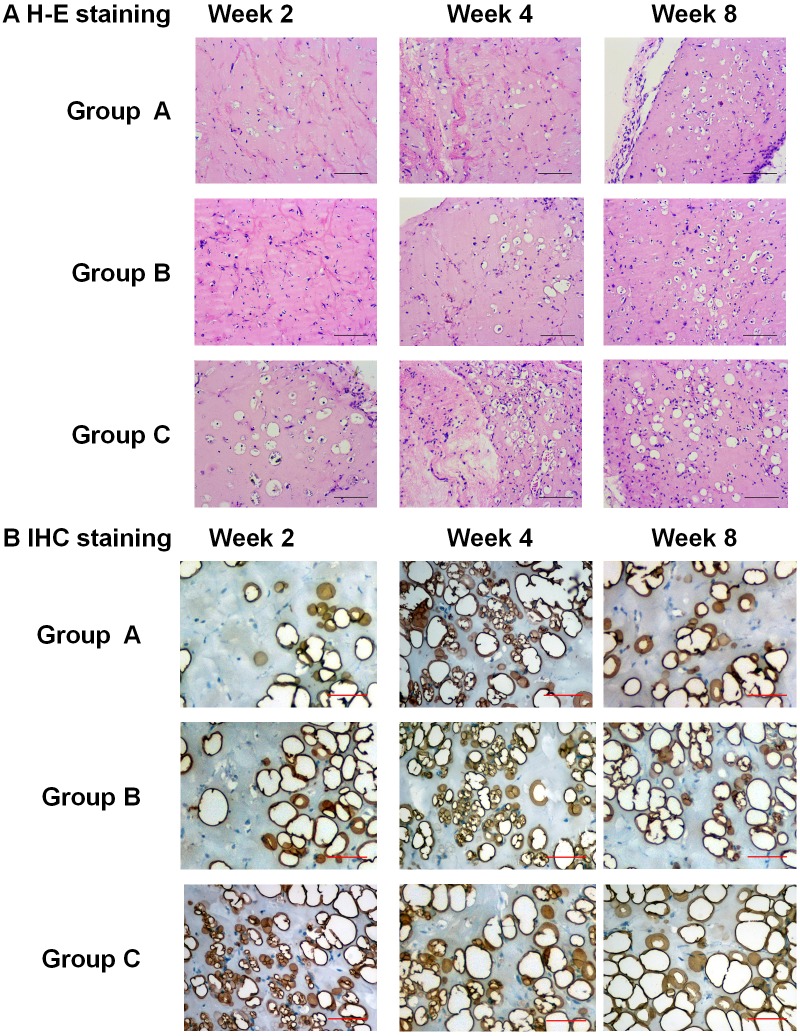
Pathological observation of adipogenic differentiation of Hem-MSCs *in vivo* **(A)** H-E staining showed the adipocytes in the plugs of all three groups. The adipogenesis in Group C (recombinational lentivirus) was obviously accelerated, compared with Group A (normal control) and Group B (non-recombinant lentivirus control). **(B)** IHC staining of perilipin A showed active adipogenesis on Week 2 in the plugs of Group C, but on Week 4 in Group A and B. Scale bar: 100μm.

H-E staining showed the adipocytes in the plugs of all three groups. In Group C, many adipocytes appeared in the plugs on Week 2, and developed into fat-like tissue on Week 4 and 8. In Group A and B, the adiocytes were rare in the plugs on Week 2, and increased on Week 4 and 8 (Figure 5A). The staining of perilipin A also showed the dense, irregular perilipin(+) adipocytes in the plugs of Group C on Week 2, which indicated the active adipogenesis. But in Group A and B, active adipogenesis was observed on Week 4 (Figure 5B). The above results suggested that the adipogenesis in Group C was earlier and stronger than that in Group A and B.

**Figure 6:**
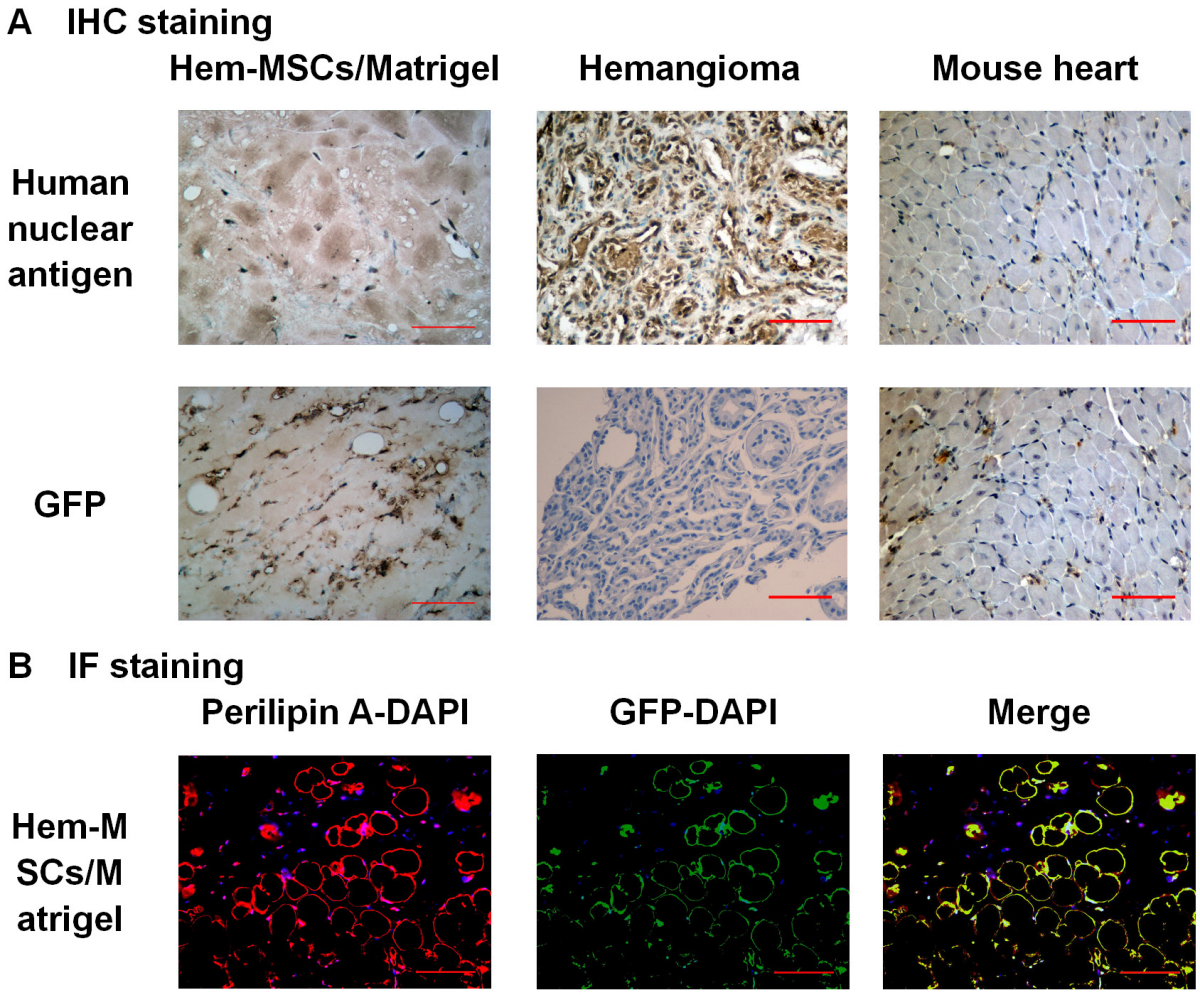
Confirmation of the origin of the cells in the plugs **(A)** IHC staining showed that human nuclear antigen was expressed by most of the cells including adipocytes in the plugs and infantile hemangioma, but not in mouse heart muscle cells. GFP was expressed in cells in the plugs, but not in IH and mouse heart muscle cells. **(B)** IF staining showed co-staining of Perilipin A and GFP in adipocytes in the plugs. Scale bar: 100μm.

In order to confirm the origin of the cells in the plugs, immunohistochemistry (IHC) staining of human nuclear antigen and immunofluorescence (IF) co-staining of Perilipin A and GFP were performed. In IHC staining, human nuclear antigen was expressed by most of the cells including adipocytes in the plugs (Figure 6A). Co-staining of Perilipin A and GFP was observed in the adipocytes in the plugs (Figure 6B). These results indicated that the cells including adipocytes in the plugs originated from the injected Hem-MSCs, not from the host mice.

**Figure 7:**
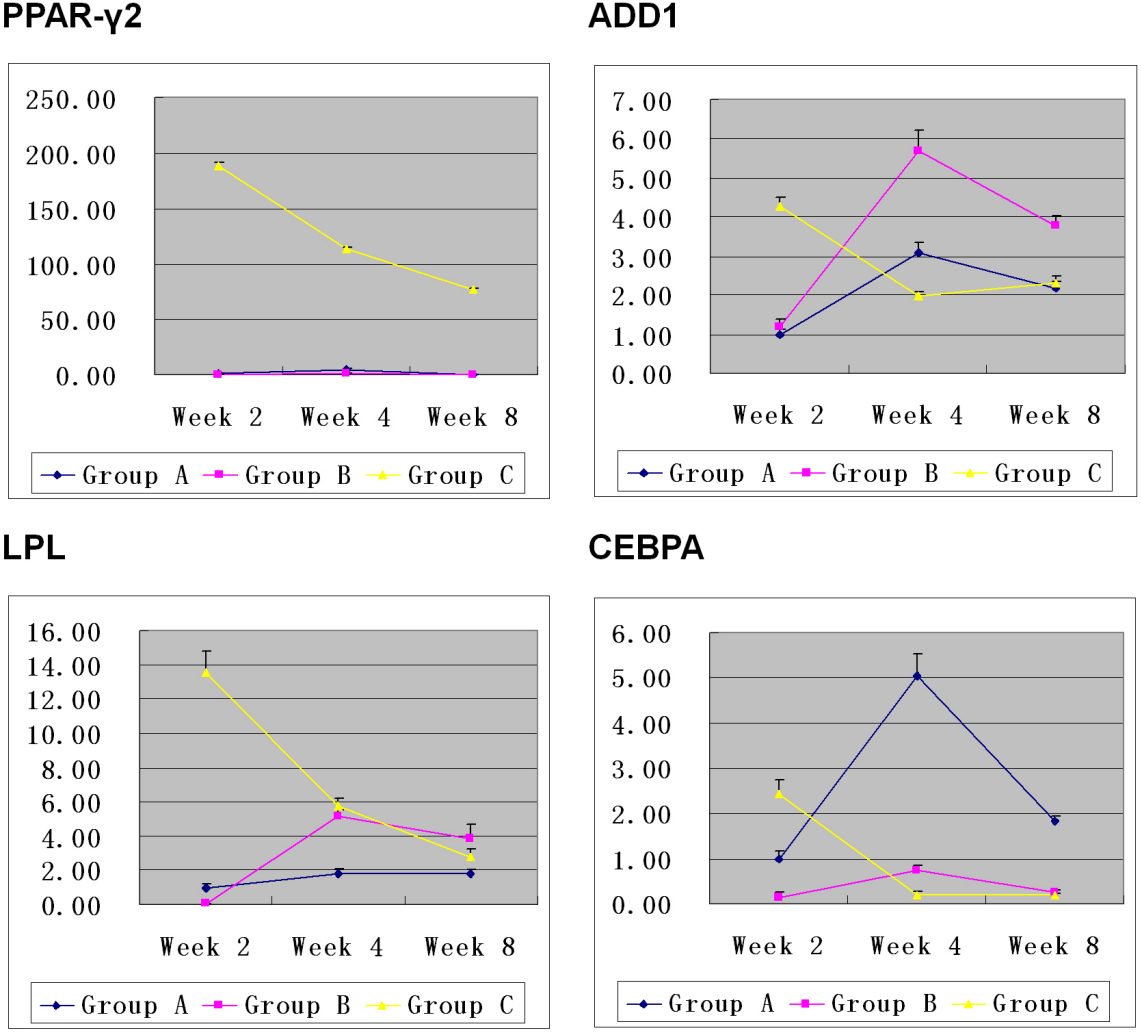
Expression of the adipogenic-related genes in Hem-MSCs/Matrigel plugs On Week 2, expression of PPAR-γ2, LPL, ADD1and CEBPA genes was obviously higher in Group C than that in Group A and B. On Week 4, genes’ expression in Group C decreased, but that in Group A and B increased. On Week 8, genes’ expression in Group C remained stable. But that in Group A and B decreased. Overall, PPAR-γ2 gene expression in Group C was consistently higher than that in Group A and B (P<0.01).

The results of QPCR showed that expression of PPAR-γ2, LPL, ADD1, and CEBPA genes in Group C on Week 2 was obviously higher than that in Group A and B. On Week 4, expression of the genes in Group C decreased while an opposite trend was observed in Group A and B. On Week 8, the genes’ expression in Group C remained stable while the gene expression in Group A and B decreased. Overall, PPAR-γ2 gene expression in Group C was constantly and obviously higher than that in Group A and B on Week 2, 4, and 8 although it kept decreasing (Figure 7).

Through comprehensive analysis of above results, we infered that pro-adipogenic genes’ expression was consistent with adipogenesis in the plugs. In Group C, the peak of adipogenesis appeared on Week 2, accompanied by the highest genes’ expression. In Group A and B, the peak of adipogenesis and the highest genes’ expression appeared on week 4 simultaneously. The results suggested that the gene expression increased in active adipogenesis while decreased at the end of adipogenesis.

## DISCUSSION

The spontaneous involution of infantile hemangioma may imply a promising new approach to treat this disease. The disappearance of microvessels and accumulation of fibrofatty tissue are two remarkable phenomena in IH's involution. Studies have shown that apoptosis of endothelial cells (ECs) may leads to the collapse and disappearance of the microvessels [5, 13]. Meanwhile adipogenesis is activated and hemangioma involutes into fibrofatty tissues finally [6–9]. So ideal therapy for IH should inhibit the angiogenesis and promote the adipogenesis in IH.

MSCs, a group of multipotential stem cells, were found to be abundent in hemangjoma and mainly reside in the perivascular region [8]. Yu Y et al first isolated MSCs from hemangioma tissue and supposed MSCs contributed to the adipogenesis in IH's involution [6]. Khan ZA et isolated CD133(+) cells from hemangioma tissue (Hemangioma-derived stem cells, Hem-SCs). Hem-SCs had the antigen profile of MSCs and show multi-lineage differentiation potential [7]. They displayed vasculogenesis/angiogenesis *in vivo* and developed into fibrofatty-like tissue finally. Above studies suggested abnormal MSCs may be the source of hemangioma. Altering the differentiation of MSCs may stop IH's growth and promote its involution.

Although we don't know exactly the mechanism of IH's involution, we speculate that MSCs’ vasculogenic potential may be inhibited by the altered expression of certain genes at some time-point (one year old in most of the children). They stop to differentiate into vascular endothelial cells, therefore the existing endothelial cells become old and go to the process of apoptosis, leading to collapse of vessels in IH. Simultaneously, MSCs’ adipogenic potential was activated and fibrofaty tissue appears. That's to say, MSCs play the leading role in the growth and involution of IH. Activation of the adipogenic potential of MSCs and induction of their differentiate into adipocytes, instead of endothelial cells, may promote IH's involution.

PPAR-γ gene is located at the p-arm of Chromosome 3, including 9 exons [14]. Based on the different promoter and splicing in the gene transcription, PPAR-γ is divided into 4 iso-forms, i.e. PPAR-γ1, PPAR-γ2, PPAR-γ3, and PPAR-γ4. Among them, PPAR-γ2 is an important nuclear transcription factor controlling the adipogenic differentiation of MSCs. Yu Y's and our studies have confirmed that PPAR-γ2 gene is expressed in Hem-MSCs [6, 8]. So we supposed that over-expression of PPAR-γ2 gene may enhance the adipogenic differentiation of Hem-MSCs. Our goal is to evaluate the possibility whether we can promote the adipogenesis in hemangioma and accelerate its involution by activating the PPAR-γ signal pathway.

In this study, we prepared the recombinant lentivirus with PPAR-γ2 gene and comfirmed that PPAR-γ2 gene is obviously up-regulated in the tranfected Hem-MSCs. In the *in vitro* tests, induction culture and Oil Red “O” staining showed adipogenic differentiation was accelerated in Hem-MSCs transfected by recombinant lentivirus. The expression of PPAR-γ2, ADD1, LPL, CEBPA genes and perilipin A protein were obviously up-regulated as well. The results indicated over-expression of PPAR-γ2 gene enhanced adipogenic differentiation of Hem-MSCs in adipogenic medium. Our previous study has reported that rosiglitazone, an agonist of PPAR-γ, could enhance adipogenic differentiation of Hem-MSCs in adipogenic midium [15]. But without adipogenic medium, rosiglitazone can't induce adipogenic differentiation. These results suggested that MSCs' adipogenic differentiation was a complex procedure controlled by various genes, instead of one singular PPAR-γ gene.

Involution and adipogenesis of IH is a spontaneous phenomenon, which suggests that the microenvironment in hemangioma can induce the adipogenic differentiation of MSCs. Our previous study showed that hemangioma-derived PDGFR-β(+) perivascular MSCs differentiated into adipocytes in an animal model [9]. In this study, we further evaluated the effect of overexpression of PPAR-γ2 gene on the *in vivo* adipogenic differentiation of Hem-MSCs. The results showed obviously accelerated adipogenesis in Hem-MSCs/Matrigel plugs with transfection by the recombinant lentivirus. Two weeks after transplantation, active adipogenesis appeared in these plugs. But in the plugs of normal control group or non-recombinant lentivirus group, the active adipogenesis was not observed until four weeks after transplantation. QPCR also showed peak expression of PPAR-γ2, ADD1, LPLand CEBPA genes on Week 2 in Hem-MSCs/Matrigel plugs with transfection by recombinant lentivirus, while on Week 4 in the plugs of normal control group or non-recombinant lentivirus group. The trend of gene expression was consistent with adipogenesis in the plugs. In active adipogenesis of Hem-MSCs, gene expression increased. At the end of adipogenesis, gene expression decreased. The *in vivo* experiment showed that over-expression of PPAR-γ2 gene upregulated the proadipogenic genes’ expression and accelerated adipogenesis. While the adipogenesis came to the end, the genes expression decreased.

In recent years, the role of PPAR-γ pathway on angiogenesis was attracting. Activation of PPAR-γ showed predominantly antiangiogenic properties, such as decreasing the expression of angiogenic factors and reducing the migration and proliferation of ECs [16, 17]. MSCs have the potential of vasculogenesis/angiogenesis [7, 18]. In fact, we observed a few of vessels in our Hem-MSCs/Matrigel plugs (Supplementary Figure 2A). But IHC staining showed only a part of vessels expressed Glut-1 (Supplementary Figure 2B). We think the vessels were from both of the differentiation of Hem-MSCs and the sprout of host mice's vessels. Because the vasculogenesis/angiogenesis in the plugs was weak, we didn't calculated the volume of micro-vessel density and made the comparative analysis. In the future work, We will isolate the endothelial cells from hemangioma and investigate the effects of PPAR-γ pathway on the proliferation, apoptosis, and migration of Hem-ECs. The results may confirm if PPAR-γ activation can inhibit the vasculogenesin/angiogenesis in IH.

## MATERIALS AND METHODS

### Isolation and culture of Hem-MSCs

Fresh hemangioma samples were obtained from Jinling Hospital and Children's Hospital in Nanjing, China, in accordance with the human subject's protocol approved by the ethics committee of Jingling Hospital (2012NZGKJ-117). Informed consent was achieved according to the Declaration of Helsinki. MSCs were separated according to the procedure described previously [6, 8]. In brief, fresh samples were rinsed in chlorhexidine solution and phosphate-buffered saline, minced, digested with 0.2% collagenase A (M9195, Sigma-Aldrich) at 37°C for one and a half hoursand then filtered through 70-μm-cell strainers (BD Falcon^TM^) to get single-cell suspension. Afterwards these cells were resuspended by Dulbecco's Modified Eagle's Medium-low glucose (DMEM-LG) (Hyclone)/10%FBS (Hyclone) supplemented with 1×PG (100U/ml penicillin, 100μg/ml gentamycin) (briefly called DMEM-LG in the passage below) and plated on plastic culture dishes (Corning). On the second day, the medium was discarded to remove the floating cells. Cells left were cultured to confluence and then divided into two groups at the ratio of 1:3.

### Preparation of the recombinant lentivirus with PPAR-γ2 gene

This procedure was performed with the help of Genechem Corp, Shanghai, China. In brief, the template of PPAR-γ2 gene was cloned from cDNA library. The primers for PPAR-γ2 gene included the forward primer (5′-AGGATCCCCGGGTACCGGTCGCCACCATGGGTGAAACTCTGGGAGATTCTCCTATTGACCCAGAAAGCGATTCCTTC-3′) and reverse primer (5′-TCCTTGTAGTCCATACCGTACAAGTCCTTG TAGATCTCCTG-3′). The size of PCR products was 1559bp, which was examined by running 5 μl products on 3% agarose gel. The recombinant lentivirus used in this study included three vectors, including GV core vector, pHelper 1.0 vector, and pHelper 2.0 vector (Figure 1A) [19]. The template of PPAR-γ2 gene was recombined into GV core vector and transformed into the competent Escherichia coli. Positive clone was confirmed by PCR, and the sequence alignment with targeted gene was accomplished. Then the GV vector was used to transfect 293T cells. The transfected cells were cultured for 36 hours, and collected to examine the expression of three flag-PPAR-γ2 fusion protein by western blot. Finally, three vectors were loaded into 293T cells. The cells were cultured for 48 hours and then collected to harvest the recombinant lentivirus.

### Detection of PPAR-γ2 gene expression in Hem-MSCs transfected by recombinant lentivirus

This test was to determine whether PPAR-γ2 gene expression was elevated in Hem-MSCs transfected by recombinant lentivirus. Three groups including Group A (DMEM-LG), Group B (non-recombinant lentivirus+ DMEM-LG) and Group C (recombinant lentivirus+DMEM-LG), were set up. The cells were cultured in six-well plates at the density of 1×10^5^ cells per well, with three wells per group. The cells were cultured in DMEM-LG for 24 hours. Then the media was replaced by another corresponding media in each group. The culture lasted for 72 hours. Then the cells were harvested for the extraction of total RNA, reverse trscription of cDNA, and detection of PPAR-γ2 gene expression by quantitative real time polymerase chain reaction (QPCR).

### *In vitro* adipogenic differentiation of Hem-MSCs

This test was to observe the effects of over-expression of PPAR-γ2 gene on *in-vitro* adipogenic differentiation of Hem-MSCs. Three groups were established as described above. The cells was seeded into the six-well plate (1×10^5^ cells per well) or 10cm dish (5×10^5^ cells per dish). The cells were cultured in the corresponding media for 72 hours. Then the media of Group B and C were replaced by the adipogenic medium. Adipogenic media included DMEM-LG /10% FBS, 5μg/ml insulin, 1μM dexamethasone, 0.5mM isobutylmethylxanthine, and 60μM indomethacin. At the 7^th^ and 14^th^ day after induction, Oil Red “O” staining was practiced on the cells in six-well plates. And the cells in 10cm dishes were harvested to detect the expression of PPAR-γ2, ADD1, LPL, and CEBPA genes by QPCR. Western blot was carried out to observe the expression of Perilipin A in the cells in 10cm dishes at the 14^th^ day after induction.

### *In vivo* adipogenic differentiation of Hem-MSCs

This test was to observe the effects of over-expression of PPAR-γ2 gene on the *in vivo* adipogenic differentiation of Hem-MSCs. This animal study was approved by the Ethics Committee of Jinling Hospital. Three groups were established as described above. Hem-MSCs were seeded into 10cm dishes (5×10^5^ cells per dish), cultured for 72 hours and harvested. Then the cells were mixed with Matrigel (356237, BD Biosciences), and injected subcutaneously into the back of 6-week-old, male athymic nude mice (n=12/group; Jinling Hospital, Nanjing, Jiangsu, China) (2×10^6^cells/300μL Matrigel per animal). The Hem-MSCs/Matrigel plugs were taken out at two, four, and eight weeks after transplantation and divided into two parts immediately. One part was fixed in 4% formaldehyde, and embedded in paraffin for H-E staining to observe the differentiation of Hem-MSCs in plugs, immunohistochemical staining of Perilipin A, GFP, and human nuclear antigen, and immunofluorescence staining of Perilipin A and GFP. The other part was frozen with liquid nitrogen and sent to detect the expression of PPAR-γ2, LPL, ADD1, and CEBPA genes by QPCR.

### Methods used in this study

#### Immuno-histochemical staining (IHC staining)

Paraffin-embedded samples were cut into sections of 5μm. Antigen retrieval was achieved by immersing them in 0.1mol/L citrate (pH 6.0) and incubating in an 800-W microwave oven for 15 minutes. Maxvision^TM^ IHC kit and DAB kit (KIT-5001 and DAB-0031, Maixin Biotech., China) were used. In brief, the sections were blocked for 30 minutes in 5% serum and incubated by the first antibodies i.e. goat anti-human Perilipin A (Ab61682, Abcam), mouse anti-human GFP (66002-1-Ig, CMC) and mouse anti-human nuclear antigen (MAB1281, Millipore) antibodies, then incubated by the second antibodies. While in the blank control group, PBS was used to replace the first antibodies.

#### Immunofluorescence staining (IF staining)

The cutting of sections and antigen retrieval was finished as described above. The sections were first incubated by goat anti-human Perilipin A antibodies (Ab61682, Abcam) with the co-staining of mouse anti-human GFP antibodies (66002-1-Ig, CMC) for 24 hours, then incubated by the second antibodies i.e. Alexa Fluor^®^ 555 donkey anti-goat IgG antibodies (A-21432, Invitrogen) or Alexa Fluor® 488 donkey anti-mouse IgG antibodies (A-21202, Invitrogen), counter-stained with DAPI (D-21490, Invitrogen). The isotype control group included goat polyclonal IgG (ab37373, Abcam) and mouse IgG1 (ab18448, Abcam). Fluorescent images were observed with fluorescence microscope, and taken photos of by digital microscope camera.

#### Quantitative real time polymerase chain reaction (QPCR)

QPCR was performed to detect the expression of PPAR-γ2, LPL, ADD1 and CEBPA genes of cells or tissues. According to the manufacturer's instructions, total RNA was extracted by Trizol total RNA isolation kit (3101-100, Shanghai Pufei Biotech Co., Ltd, China). Then reverse transcription was done to get cDNA with M-MLV RT kit (M1705, Promega). QPCR was carrid out with SYBR Master Mixture (DRR041B, TAKARA) and other reagents on a real time PCR machine (LightCycler480, Roche). The primers for PPAR-γ2, LPL, ADD1, CEBPAand internal control GAPDH genes were showed in Table 1. The two-step PCR reactions were performed with the following temperature settings: 95°C for 30 seconds; then 95°C for 5 seconds and 60°C for 30 seconds, 40 cycles; and 95°C for 15 seconds, 60°C for 30 seconds, 95°Cfor 15 seconds. Size of the PCR products was examined by running 5 μl products on 3% agarose gel. In the analysis of data, the relative expression of genes was showed as 2^−ΔΔCt^ (ΔCt = Ct_tageted gene_ − Ct_internal gene_; -ΔΔCt =ΔCt_contral group_ − ΔCt_experimental group)_.

**Table 1:** The primers of PPAR-γ2, LPL, ADD1, CEBPA, and GAPDH genes

Gene	Forward primer	Reverse primer	Size of products (bp)
PPAR-γ2	AGAAAGCGATTCCTTCACTGAT	AGAATGGCATCTCTGTGTCAAC	80
LPL	AATGTACCTGAAGACTCGTTCT	GTTCTCCAATATCTACCTCTGTG	224
ADD1	CCTTGGAGAAGTGGCTTATCAT	GGATTCAGCAGGACTAAGTTGT	246
CEBPA	CCAGAAAGCTAGGTCGTGGGT	TGGACTGATCGTGCTTCGTGT	174
GAPDH	TGACTTCAACAGCGACACCCA	CACCCTGTTGCTGTAGCCAAA	121

#### Western blot

Cells were lysed with RIPA Buffer (P0013C, Beyotime, Shanghai, China), submitted to sodium dodecyl sulfate–polyacrylamide-gel electrophoresisand transferred to a membrane. The membranes were then incubated by goat-anti-human Perilipin A antibodies (Ab61682, Abcam) and mouse anti-β-actin antibodies (sc-130301, Santa Cruze), and then incubated by rabbi-anti-goat IgG H&L (HRP) (ab6741, Abcam) and rabbit anti-mouse IgG H&L (HRP) (ab6728, Abcam). Chemiluminescent sensitive film (32106, Pierce™ ECL Western Blotting Substrate, Thermo Scientific) was used to detect antigen-antibody complexes. The electrophorograms were semi-quantitatively analyzed by the software “Image J”.

### Statistics

Statistics were processed with Microsoft^®^ Office Excel 2007. Data was presented as mean value or mean value ± SD and analyzed using Student's two-tailed *t* test where appropriate. Differences were considered significant at *P*<0.05.

## CONCLUSIONS

In this study, the recombinant lentivirus with PPAR-γ2 gene was prepared and used to transfect Hem-MSCs. *In-vitro* and *in-vivo* tests showed that over-expression of PPAR-γ2 gene enhanced and accelerated the adipogenic differentiation of Hem-MSCs, accompanied by up-regulation of adipogenic-related genes. The results may provide preliminary evidences for the targeted therapy of IH by activating adipogenesis and promoting involution via PPAR-γ pathway, such as intra-lesional injection or topical application of rosiglitazone. However, the adipogenic differentiation of Hem-MSCs in animal model may not be the same as that in hemangioma. So the significance of this study on clinical therapy needs further investigation.

## SUPPLEMENTARY MATERIALS FIGURES



## Abbreviations

IH: infantile hemangioma
MSCs: mesenchymal stem cells
Hem-MSCs: hemangioma-derived mesenchymal stem cells
PPAR: peroxisome proliferator-activated receptor
RXR: retinoid X receptor
DMEM-LG: Dulbecco's Modified Eagle's Medium-low glucose
GFP: green fluorescent protein
IHC: immunohistochemistry
IF: immunofluorescence
QPCR: quantitative real time polymerase chain reaction
CD: cluster of differentiation
