# Supplementary material for: Over-expression of PPAR-γ2 gene enhances the adipogenic differentiation of hemangioma-derived mesenchymal stem cells in vitro and in vivo
Source: Oncotarget. 2017 Dec 26;8(70):115817–28. doi: 10.18632/oncotarget.23705 (PMC5777815; doi:10.18632/oncotarget.23705)
Supplement: Supplementary file 1 [file oncotarget-08-115817-s001.pdf]

Over-expression of PPAR-γ2 gene enhances the adipogenic differentiation of hemangioma-derived mesenchymal stem cells *in vitro* and *in vivo*

SUPPLEMENTARY MATERIALS

CGTTTTTGGCTTTTTTGTTAGACGAAGCTTGGGCTGCAGGTCGACTCTAGAGGATCCCCGGGTACCGGTCG
CCACCATGGGTGAAACTCTGGGAGATTCTCCTATTGACCCAGAAAGCGATTCCTTCACTGATACACTGTCTGC
AAACATATCACAAGAAATGACCATGGTTGACACAGAGATGCCATTCTGGCCCACCAACTTTGGGATCAGCTC
CGTGGATCTCTCCGTAATGGAAGACCACTCCCACTCCTTTGATATCAAGCCCTTCACTACTGTTGACTTCTCCA
GCATTTCTACTCCACATTACGAAGACATTCCATTCACAAGAACAGATCCAGTGGTTGCAGATTACAAGTATGA
CCTGAAACTTCAAGAGTACCAAAGTGCAATCAAAGTGGAGCCTGCATCTCCACCTTATTATTCTGAGAAGAC
TCAGCTCTACAATAAGCCTCATGAAGAGCCTTCCAACTCCCTCATGGCAATTGAATGTCGTGTCTGTGGAGAT
AAAGCTTCTGGATTTCACTATGGAGTTCATGCTTGTGAAGGATGCAAGGGTTTCTTCCGGAGAACAATCAGA
TTGAAGCTTATCTATGACAGATGTGATCTTAACTGTCGGATCCACAAAAAAAGTAGAAATAAATGTCAGTACT
GTCGGTTTCAGAAATGCCTTGCAGTGGGGATGTCTCATAATGCCATCAGGTTTGGGCGGATGCCACAGGCC
GAGAAGGAGAAGCTGTTGGCGGAGATCTCCAGTGATATCGACCAGCTGAATCCAGAGTCCGCTGACCTCCG
GGCCCTGGCAAAACATTTGTATGACTCATACATAAAGTCCTTCCCGCTGACCAAAGCAAAGGCGAGGGCGAT
CTTGACAGGAAAGACAACAGACAAATCACCATTCGTTATCTATGACATGAATTCCTTAATGATGGGAGAAGAT
AAAATCAAGTTCAAACACATCACCCCCCTGCAGGAGCAGAGCAAAGAGGTGGCCATCCGCATCTTTCAGGG
CTGCCAGTTTCGCTCCGTGGAGGCTGTGCAGGAGATCACAGAGTATGCCAAAAGCATTCCTGGTTTTGTAAA
TCTTGACTTGAACGACCAAGTAACTCTCCTCAAATATGGAGTCCACGAGATCATTTACACAATGCTGGCCTCC
TTGATGAATAAAGATGGGGTTCTCATATCCGAGGGCCAAGGCTTCATGACAAGGGAGTTTCTAAAGAGCCT
GCGAAAGCCTTTTGGTGACTTTATGGAGCCCAAGTTTGAGTTTGCTGTGAAGTTCAATGCACTGGAATTAGA
TGACAGCGACTTGGCAATATTTATTGCTGTCATTATTCTCAGTGGAGACCGCCCAGGTTTGCTGAATGTGAAG
CCCATTGAAGACATTCAAGACAACCTGCTACAAGCCCTGGAGCTCCAGCTGAAGCTGAACCACCCTGAGTC
CTCACAGCTGTTTGCCAAGCTGCTCCAGAAAATGACAGACCTCAGACAGATTGTCACGGAACACGTGCAGC
TACTGCAGGTGATCAAGAAGACGGAGACAGACATGAGTCTTCACCCGCTCCTGCAGGAGATCTACAAGGAC
TTGTACGGTATGGACTACAAGGATGACGATGACAAGGATACAAAGACGACGA

Supplementary Figure 1: Gene sequencing of the transformants of positive clones of competent E. Coli. The sequence alignment showed 100% accordance with targeted gene, PPAR-γ2.

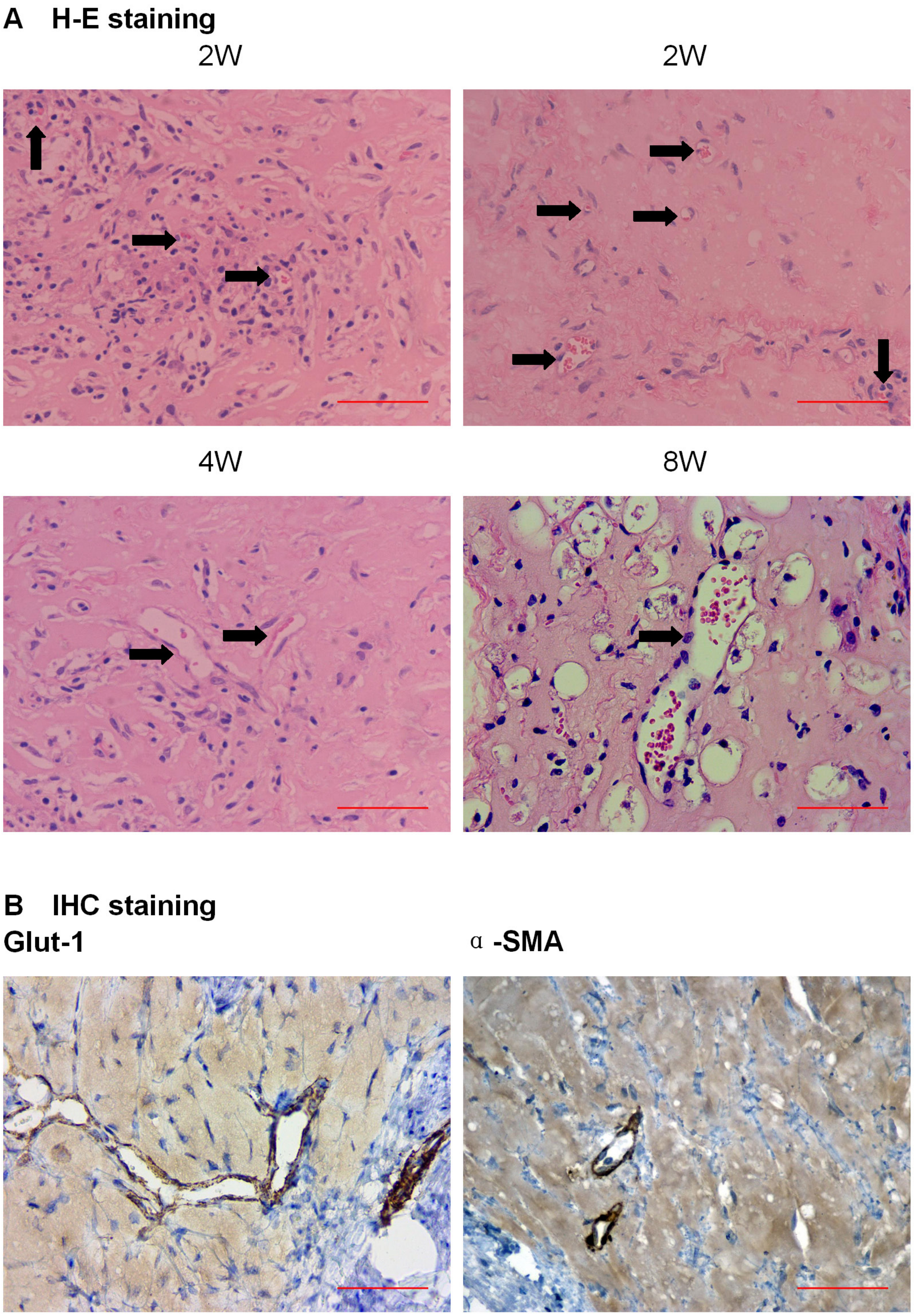

Supplementary Figure 2: The vasculogenesis/angiogenesis in Hem-MSCs/Matrigel plugs. **(A)** In H-E staining, a few of vessels appeared in the Hem-MSCs/Matrigel plugs (black arrows) in which there were red cells. **(B)** In IHC staining, Glut-1 (a specific marker antigen of hemangioma endothelium) and α-SMA were expressed on the vessel wall. Scale bar:100μm.
